# Sub-paresthesia spinal cord stimulation reverses thermal hyperalgesia and modulates low frequency EEG in a rat model of neuropathic pain

**DOI:** 10.1038/s41598-018-25420-w

**Published:** 2018-05-08

**Authors:** Suguru Koyama, Jimmy Xia, Brian W. Leblanc, Jianwen Wendy Gu, Carl Y. Saab

**Affiliations:** 1 Department of Neurosurgery and Department of Neuroscience, Rhode Island Hospital & Brown University, Providence, RI USA; 2 Laboratory for Pharmacology, Asahi Kasei Pharma Corporation, Mifuku, Shizuoka Japan; 3 Boston Scientific Neuromodulation, Valencia, CA USA

## Abstract

Paresthesia, a common feature of epidural spinal cord stimulation (SCS) for pain management, presents a challenge to the double-blind study design. Although sub-paresthesia SCS has been shown to be effective in alleviating pain, empirical criteria for sub-paresthesia SCS have not been established and its basic mechanisms of action at supraspinal levels are unknown. We tested our hypothesis that sub-paresthesia SCS attenuates behavioral signs of neuropathic pain in a rat model, and modulates pain-related theta (4–8 Hz) power of the electroencephalogram (EEG), a previously validated correlate of spontaneous pain in rodent models. Results show that sub-paresthesia SCS attenuates thermal hyperalgesia and power amplitude in the 3–4 Hz range, consistent with clinical data showing significant yet modest analgesic effects of sub-paresthesia SCS in humans. Therefore, we present evidence for anti-nociceptive effects of sub-paresthesia SCS in a rat model of neuropathic pain and further validate EEG theta power as a reliable ‘biosignature’ of spontaneous pain.

## Introduction

Neuropathic pain is highly prevalent, costly and contributes to the opioid crisis^1,2^, in part due to ineffective pharmacotherapy and overprescription of opioids^3,4^. Spinal cord stimulation (SCS) is a non-opioid, FDA-approved therapy for severe pain conditions^5,6^. Though SCS is known to reduce pain severity by more than 50% in more than 50% of patients, there is urgent need for achieving better clinical outcomes.

Paresthesia secondary to SCS challenges the gold standard double-blind study design because it is impossible to blind the subjects, thus undermining research efforts to enhance the therapeutic benefits of SCS. Interestingly, it was recently demonstrated that SCS at current amplitudes below those evoking sensory perception (i.e. sub-paresthesia SCS) is effective in alleviating pain^7^, and offers the benefit of reducing subjective bias and placebo effects in prospective studies. Therefore, there has been an emerging interest in establishing empirical criteria for sub-paresthesia SCS and in elucidating its basic mechanisms of action at supraspinal levels.

In this study, we used a rat model of neuropathic pain to test our hypothesis that sub-paresthesia SCS attenuates behavioral signs of neuropathic pain, and modulates pain-related ‘biosignature’ activity in primary somatosensory cortex (S1), which mediates the discrimination and localization of pain^8–10^. Briefly, a principle feature of the biosignature consists of electroencephalography (EEG) power enhancement in the theta band (4–8 Hz) that is correlated positively with nociceptive behavior and negatively with analgesic treatment in awake, unrestrained rodents^11–14^.

In SCS studies using pre-clinical animal models, current amplitudes are mostly determined under anesthesia in reference to motor thresholds (i.e. minimal current amplitude eliciting muscle twitch), which vary widely between animals and are incompatible with clinical settings whereby SCS protocols are based on the verbal report of paresthesia (i.e. paresthesia-based). To overcome this limitation, we determined paresthesia thresholds in rats according to behaviors indicative of alertness to SCS in awake states. We chose 50 Hz, 200 µs pulse width as standard SCS parameters that have been used clinically for over 50 years, while setting the amplitude according to perception threshold (defined as overt behavioral response to the onset of SCS, see Methods).

Epidural SCS leads were chronically implanted and stimulation was applied continuously for 3–4 hrs during awake state. Data show that rats with behavioral signs of neuropathic pain manifest increased S1 theta power, confirming our previous reports^11–14^. Sub-paresthesia SCS attenuated nociceptive behavior and power amplitude in the 3–4 Hz range, but not the 5–8 Hz range of the theta band. These results are consistent with clinical data showing significant yet modest analgesic effects of sub-paresthesia conventional (50 Hz) SCS on spontaneous pain in humans^7^ (however see Kapural *et al*. for a claim with regards to superior efficacy of sub-paresthesia SCS at 10 kHz^15^). Thus, we provide novel evidence for anti-nociceptive effects of sub-paresthesia SCS in a rat model of neuropathic pain. We further validate EEG theta power as a ‘biosignature’ of spontaneous pain in pre-clinical, and potentially clinical studies testing the efficacy of neuromodulation technologies for pain management.

## Results

**Figure 1:**
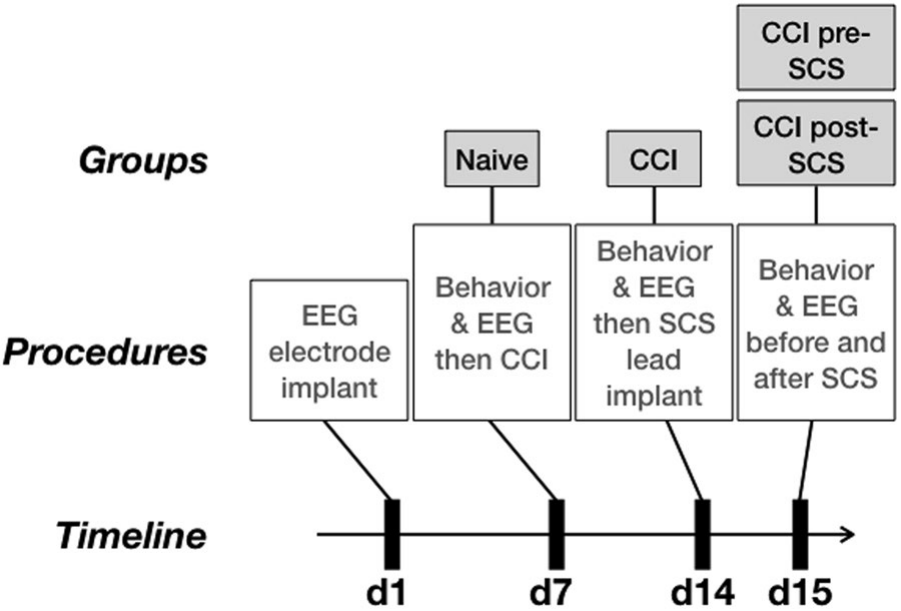
Experimental design and timeline: EEG electrodes were implanted on day 1, CCI was induced contralateral to S1 electrode on day 7, an SCS lead was implanted on day 14 (i.e. d7 CCI) and SCS was applied continuously for 3–4 hrs on day 15. Behavioral testing and EEG recordings were performed on day 7 prior to CCI (Naive group), day 14 prior to SCS lead implant (CCI group), and on day 15 before and after SCS (pre-SCS group and post-SCS group, respectively).

**Figure 2:**
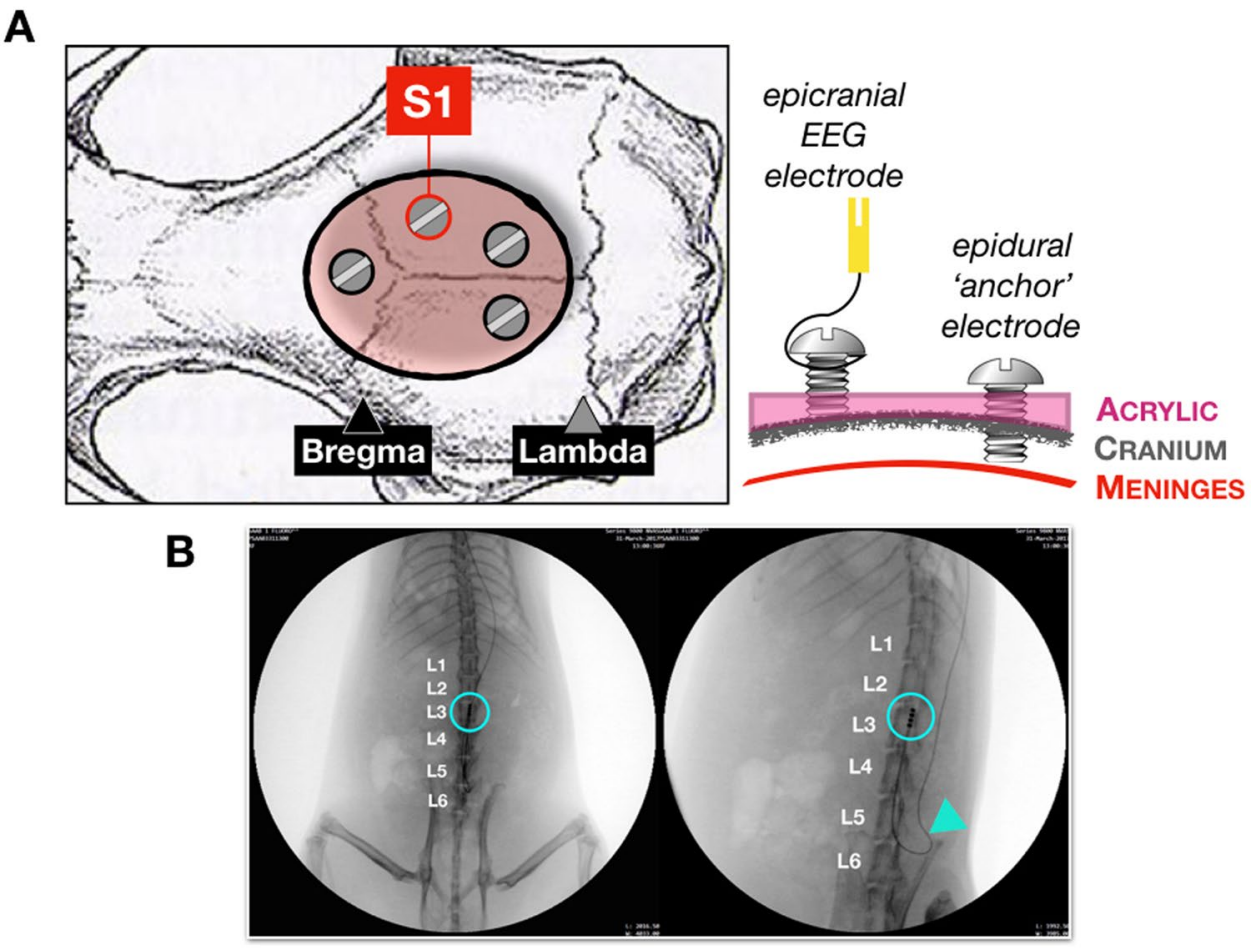
(**a**) Electrode montage: Three epidural screws for anchor and one epicranial screw for EEG recording were placed over brain structures determined stereotaxically (Bregma 2.2, 2 mm lateral for S1; red circle). Tungsten wires were wrapped around the EEG screw and attached to a pin connector. All screws were then covered in a pool of acrylic poured in a cylindrical plastic mold to form the implant’s base structure. The mold was later removed and the skin was sutured. (**b**) Fluoroscopy: Representative X-ray images showing epidural placement of the SCS lead (blue circle) at spinal L2-3 level. Arrow indicates point of exit of the lead from the spine and attachment to the vertebral body. Lead then migrated subcutaneously towards the second point of exit from the body at a cervical level, where it was protected by a ‘jacket’.

**Figure 3:**
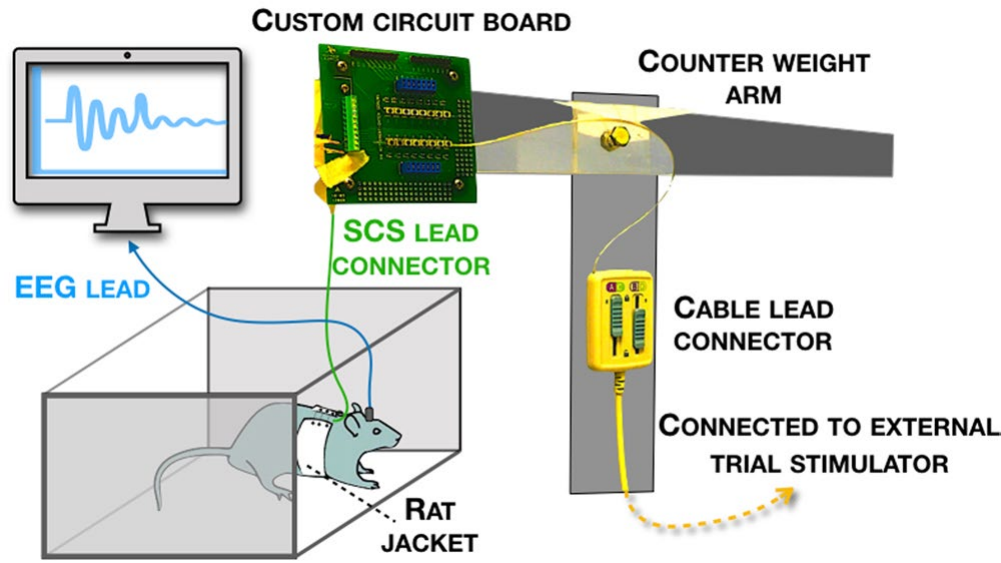
Housing & testing unit: The open-top acrylic cage allowed rats to move around freely without worrying about the lead and tubing pulling at the implant site. An acrylic platform and pole held the swivel and counterweight device. The counterweight system comfortably eliminated slack along the tubing to the stimulator. This custom-built system allowed for continuous SCS and EEG recording in awake rats.

In the experiments described below, behavioral and electrophysiological data were recorded in awake rats (n = 9); of these, two rats were excluded from EEG analysis due to electrode failure (see Figs 1–3 and Methods for detailed description of the experimental set-up and procedures). Values are reported as mean ± standard error (SE), unless otherwise stated as ± standard deviation (SD).

**Figure 4:**
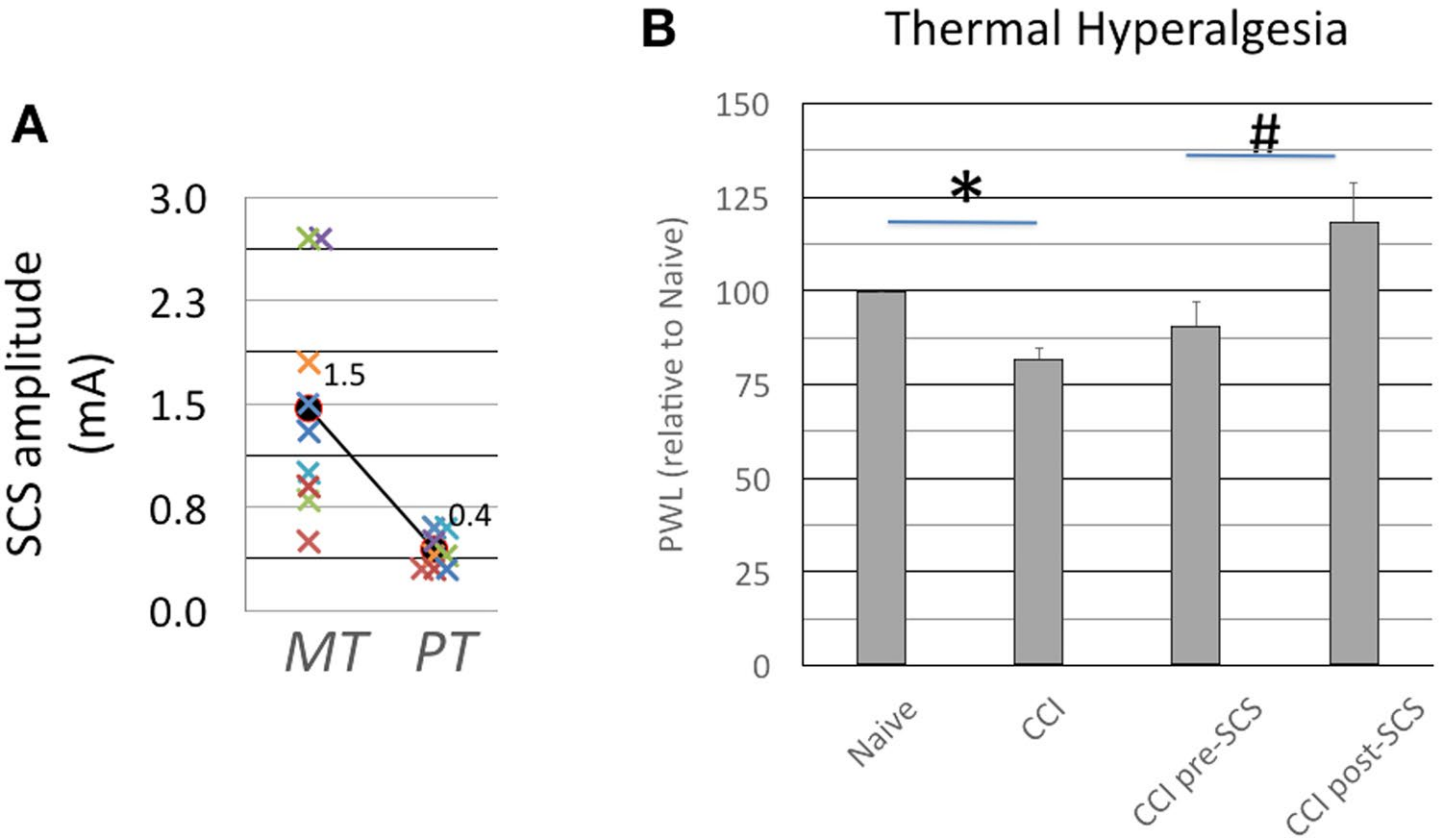
(**a**) Perception threshold versus Motor threshold: Motor threshold (MT) was determined under anesthesia (2.5% isoflurane) and varied widely ([0.5–2.7] mA, $\bar{{\rm{x}}}$ = 1.42 ± 0.75 mA, n = 9 rats) compared to perception threshold (PT), which was determined in awake rats and showed more consistent results ([0.3–0.6] mA, $\bar{{\rm{x}}}$ = 0.44 ± 0.12 mA). In each rat, SCS was continuously delivered for 3–4 hrs at PT-01 mA ($\bar{{\rm{x}}}$ = 75.5% ± 7.3 PT). (**b**) Thermal hyperalgesia: Paw withdrawal latencies (PWL) showed significantly decreased latencies following CCI compared to baseline naive (*p < 0.05, n = 9 rats). However, 3–4 hrs SCS at sub-paresthesia current amplitude (i.e. PT-0.1 mA) significantly reversed PWL, suggesting attenuation of thermal hyperalgesia (^#^p < 0.05, n = 9 rats).

Motor threshold (MT) and perception threshold (PT) were determined under anesthetized (2.5% isoflurane) and awake conditions, respectively. As shown in Fig. 4a, MT varied widely (1.42 mA ± 0.75 SE, 2.25 SD, coefficient of variation 158, n = 9 rats), compared to PT which was more consistent (0.44 mA ± 0.12 SE, ± 1.42 SD, coefficient of variation 82), and comparably similar to a previous study^15^. After determination of MT and PT, each rat was continuously stimulated for 3–4 hrs at PT – 0.1 mA in a custom-built housing and testing unit, allowing rats to move freely (Fig. 3), followed immediately by EEG recording and behavioral assessment.

The results of behavioral assessment are summarized in Fig. 4b. Relative to naive (100%), paw withdrawal latencies (PWL) following CCI significantly decreased to 81.7% ± 3.3 (n = 9 rats), suggesting symptoms of thermal hyperalgesia. After 3–4 hrs of SCS at sub-paresthesia current amplitude, PWL was significantly increased to 118% ± 11 relative to pre-SCS (90.6% ± 6.8). Mean values for PWL were 9.68 ± 0.45 s, 7.72 ± 0.30 s, 9.05 ± 0.51 s and 11.12 ± 0.78 s for Naive, CCI, pre-SCS and post-SCS, respectively.

**Figure 5:**
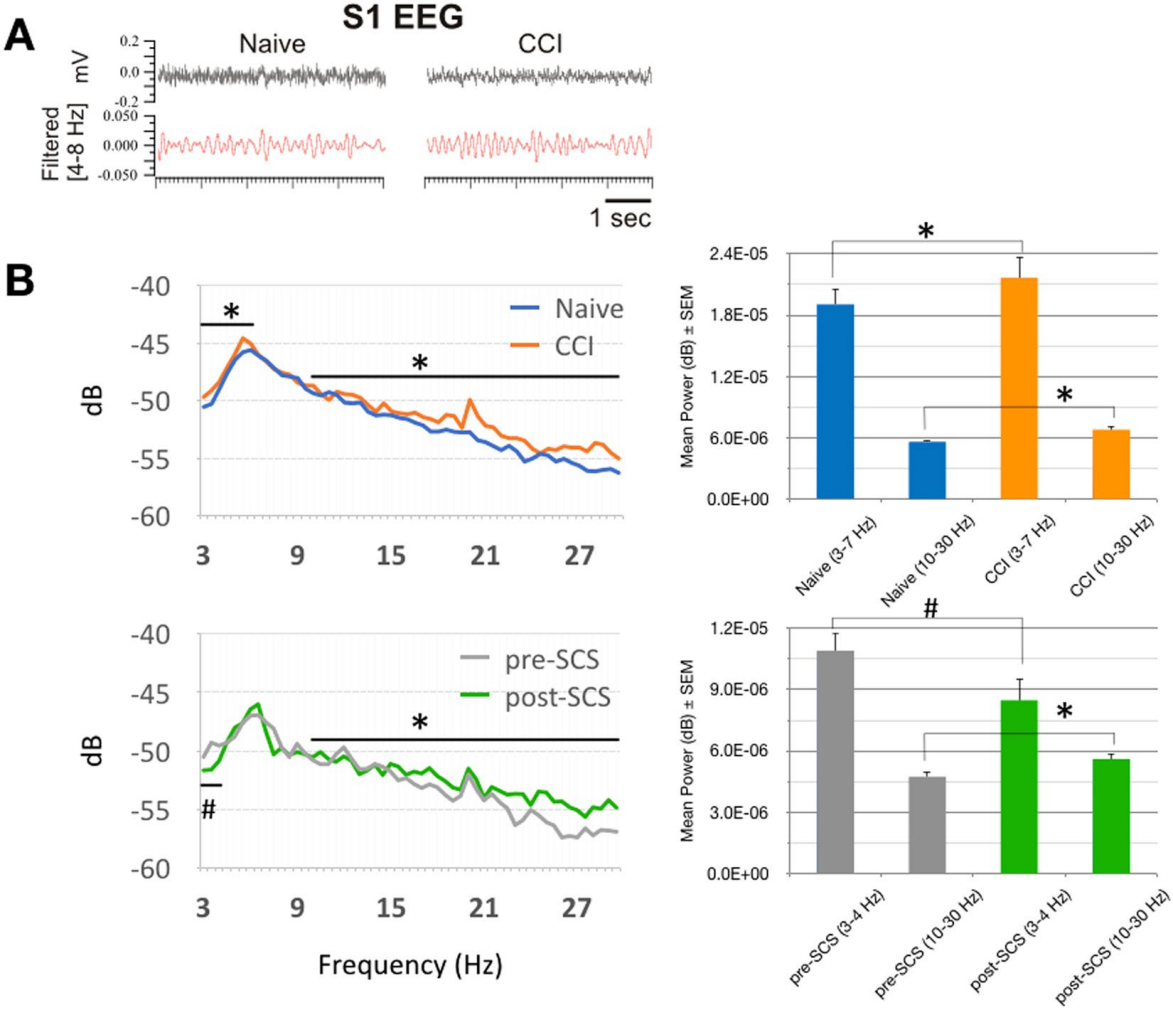
(**a**) EEG waveforms: Traces show representative EEG recordings from one rat under naive (baseline) conditions and at seven days after chronic constriction injury (CCI). Black traces show original waveforms (16.6 kHz) from S1 electrode contralateral to CCI, red traces show corresponding band-pass filtered waveforms in the theta band (4–8 Hz). Note increased theta oscillations in CCI. (**b**) EEG S1 Power: Power spectral densities (3–30 Hz) in naive rats, CCI, pre-SCS, and post-SCS in the same rats (dB = 10 × Log_10_ (mV^2^)). Note significant increase in power in the theta band (3–7 Hz) and between 10–30 Hz after CCI (*p < 0.05, n = 7 rats). SCS significantly attenuated power within 3–4 Hz (^#^p < 0.05, n = 7 rats) while further increasing power between 10–30 Hz (*p < 0.05, n = 7 rats).

Artifact-free EEG waveforms were recorded in unrestrained rats during periods of idle behavior (Fig. 5a). Overall, EEG power was significantly enhanced in the low frequency theta band (3–7 Hz) seven days after CCI (2.16E-05 mV^2 ± 1.99E-06 compared to naive 1.90E-05 mV^2 ± 1.54E-06; Fig. 5b), as expected based on previous studies using the same animal model^12–14^. Concomitantly, EEG power in the 10–30 Hz band was significantly increased after CCI (6.82E-06 mV^2 ± 2.92E-07 compared to naive 5.61E-06 mV^2 ± 2.33E-07). After SCS, EEG power was significantly decreased in the 3–4 Hz range (8.47E-06 mV^2 ± 1.04E-06 compared to pre-SCS 10.89E-06 mv^2 ± 0.85E-06), suggesting partial reversal of pain-related theta enhancement, and parallel to a reversal in the behavioral signs of thermal hyperalgesia. Interestingly, EEG power in the 10–30 Hz range was further increased (5.60E-06 mV^2 ± 2.74E-07 compared to pre-SCS 4.74E-06 mV^2 ± 2.58E-07), indicating that modulation of EEG within this band is not related to nociceptive behavior.

## Discussion

The primary aim of this study was to test the hypothesis that continuous (3–4 hrs) sub-paresthesia SCS (50 Hz, 200 µs) in awake rats attenuates behavioral signs of neuropathic pain. We also sought to elucidate the mode of action of SCS at a cortical level using a ‘biosignature’ of spontaneous pain consisting of increased EEG theta power. Results show that sub-paresthesia SCS effectively reverses thermal hyperalgesia in rats with CCI, and significantly reduces EEG power over S1 in the 3–4 Hz range, which overlaps with the theta band (4–8 Hz).

Our experiments were carefully designed to be translational and to have more direct clinical relevance compared to prior pre-clinical studies. Our experimental approach was unique in many ways, including: 1) Use of paresthesia threshold to determine current amplitudes, which is more clinically relevant compared to motor threshold and leading to less variability in SCS parameters between animals; 2) continuous SCS in awake animals, which ruled out anesthetic effects; 3) pain assessment using behavioral testing and EEG as complementary methods to evaluate stimulus-dependent (hyperalgesia) and stimulus-independent (EEG theta) sensory states indicative of pain, while elucidating the supraspinal mechanisms of SCS.

First, our results show that paresthesia thresholds were fairly consistent in all rats, in contrast to motor thresholds that varied widely, arguably due to anesthesia. Accordingly, it is assumed that all rats in this study received comparable electrical charges per second. With the exception of two studies documenting the use of continuous SCS in awake rats *via* chronically implanted leads^16,17^, most studies using animal pain models apply SCS during anesthesia^18–20^. Of note, the use of paresthesia threshold in rats has been previously reported, but SCS was applied at a higher frequency (500, 1,000 and 10,000 Hz)^16^ compared to this study (50 Hz).

Second, SCS significantly attenuated thermal hyperalgesia at day 7 after CCI. Thermal hyperalgesia and mechanical allodynia, commonly used methods that are considered cardinal signs of neuropathic pain in awake rats, are stimulus-dependent reflex behaviors indicative of hypersensitivity. However, the primary outcome and gold standard of pain assessment in clinical studies is the patient’s verbal report of spontaneous or stimulus-independent pain, not hypersensitivity. Hence, we predicted that EEG in non-restrained awake rats, using theta power as a valid correlate of spontaneous pain, is more directly relevant to the clinical setting. We previously demonstrated that theta (4–8 Hz) power is correlated positively with nociceptive behaviors and negatively with analgesic treatments in rodent models of pain^11–13^. We further elucidated the relevant thalamocortical circuits modulating theta power in nociceptive states^14^, showed evidence of increased theta power in humans evoked by a noxious cold stimulus^21^, and proposed a predictive, hierarchical model of functional connectivity in the brain as a theoretical framework for pain-related theta power^22^. Accordingly, data obtained in this study showing power reduction in the 3–4 Hz band suggest that SCS may not result in a complete reduction of spontaneous pain, corroborating results of a recent human study^7^. Though it has been generally assumed that patients must feel the paresthesias of SCS in order to obtain effective pain relief, sub-paresthesia SCS has been shown to confer analgesia in patients with refractory angina pectoris^23^ or other neuropathic pain conditions^7^ as measured by the visual analogue scale, albeit effects are significantly less compared to conventional paresthesia SCS (however, see^24^ using 10 kHz SCS). Interestingly, our data showed enhancement of power in the 10–30 Hz range in rats with CCI and a further increase following SCS. Whereas the perceptual correlate of power modulation within the beta and low-gamma bands remains to be elucidated (see^25^ for phase-locking between theta and gamma oscillations, and^26^ for a discussion of the relation between gamma oscillations and pain), we conclude that modulation of these bands is not relevant to the analgesic effects of sub-paresthesia SCS. While we do note that the lead implant resulted in a uniform reduction of power in the 3–30 Hz range, behavioral pain sensitivity was not changed. Hence, the putative effects of the lead implant *per se* did not constitute a confounding variable with regards to our interpretation of the effects of SCS. Collectively, our results confirm the validity of EEG theta power as a reliable biomarker of spontaneous pain and as a predictor of effective *versus* ineffective analgesia.

Third, the effects of SCS on brain activity is incompletely understood^27^. In a systematic review of the literature, it was concluded that empirical evidence for supraspinal effects of SCS is limited and inconclusive^27^. This conclusion was based on studies using functional imaging and evoked EEG or MEG responses (which is different from resting state EEG). Although SCS initially emerged as a direct application of the Gate Control Theory at a spinal level, analgesic effects must manifest eventually *via* ascending input to the brain. Consequently, our scientific understanding of the supraspinal mechanisms of SCS analgesia remains severely restricted. For example, the mechanisms of sub-paresthesia, high frequency SCS is attributed primarily to spinal cord segmental (‘at-level’) mechanisms^28^. Based on our results, however, it is clear that sub-paresthesia SCS modulates activity in S1 which mediates the discrimination and localization of pain^8–10^.

In conclusion, our experimental paradigm and approach in this study are fundamentally aligned with clinical settings, a major advantage for pre-clinical transitional studies and prospective double-blind clinical studies.

## Methods

### Animals

Adult male Sprague-Dawley rats were housed individually, under a 12-hour light/dark cycle, in a temperature and humidity controlled environment with food and water available ad libitum. All procedures were approved by the Rhode Island Hospital Institutional Animal Care and Use Committee. All surgical procedures were performed under deep anesthesia (isoflurane, 2–2.5%). Buprenorphine (30 mg/kg, i.p.) was administered once daily for 3 consecutive days for post-operative pain relief secondary to EEG implant and CCI, and 1 dose only on the day of SCS lead implant. All the methods were carried out in accordance with the relevant guidelines and regulations and experiments were approved by the Rhode Island Hospital Institutional Animal Care and Use Committee.

### Electrode Montage

Head was fixed in a stereotaxic apparatus. A small skin incision was used to expose the skull (Fig. 2a). For EEG montage 1 stainless steel ‘screw’ electrode was placed over intact skull corresponding to S1 hindlimb area without craniotomy (Bregma −2.2, 2 mm mediolateral). Three stabilization screws were placed after minimal craniotomies using a drill to anchor the EEG electrode chronically using dental acrylic. The EEG screw was tethered to a silver wire attached to a female miniature pin connector (A&M Systems, Sequim, WA). Reference electrode consisted of a silver wire threaded to the skin at the back of the neck.

### CCI Pain Model

One week after EEG implant, chronic constriction injury (CCI) was induced as a model of chronic neuropathic pain^29^. Rats were anesthetized with 2.5% isoflurane and the common sciatic nerve was exposed by separating the surrounding muscles and then loosely ligated with 3 chromic gut sutures contralateral to the EEG electrode over S1.

### SCS lead implant

A cylindrical four-contact stimulating electrode (0.72 mm diameter, provided by Boston Scientific Neuromodulation, Valencia, CA, USA) was inserted along the midline epidural space and advanced to the L2-3 vertebral level (Fig. 2b). The lead was then secured to the spinous process to prevent migration and tunneled subcutaneously to exit from the animal’s neck and protected in a jacket. A customized system compromising a rat jacket (Harvard Apparatus) to house the lead up to an adapter circuit board and a External Trial Stimulator (ETS; provided by BSC) was built in the Saab lab (Fig. 3). The ETS and circuit board were suspended by a counterweight system that allowed free movement of the rat. SCS was applied for 3–4 hr on d7 after CCI (24 hr after lead implant). EEG and behavioral data (see below) were collected immediately after SCS was terminated.

### Electrical Stimulation

To investigate the relation between motor threshold (MT) and perception threshold (PT), current was increased by 0.1 mA increments in anesthetized animals (2.5% isoflurane) until reaching motor threshold (MT) (50 Hz, 200 µs, biphasic with passive charge recovery), defined as a reflex response in the tail or hindlimb. Animals were then allowed to recover from anesthesia, and current amplitude was increased by 0.1 mA increments starting at 0.1 mA until perception threshold (PT) was reached (defined as overt behavioral response to the onset of SCS, for example attending to hindlimb or lower body, or alertness directly related to SCS onset). SCS amplitude was finally set at PT *minus* 0.1 mA, and rats were stimulated for the following 3–4 hr continuously. During this time, rats were housed in an open-top acrylic housing unit such that free movement was possible.

### Electrophysiological Recording

Before EEG recording, pin connectors from each electrode were tethered to preamplifier headstages leading to a multichannel amplifier (iso- DAM8A; WPI Inc, Sarasota, FL). Amplification for each channel was set at x1000. This system allowed free movement of tethered rats with no head restraint while recording EEG signals simultaneously from S1. Rats could freely navigate individually in Plexiglas chambers. The rat’s behavior was visually monitored, noting periods of rest. Each EEG recording session was approximately 5 minutes per animal. Of that 5-min interval, 3 × 10-sec epochs were selected randomly during rest state per condition, per animal. After 15 minutes of acclimation, EEG waveforms were sampled at 16.6 kHz and down- sampled offline to 250 Hz. Only data during awake resting periods (defined as idle wakefulness with no locomotor behavior) were further analyzed. Potentials generated due to vigorous myogenic activity (such as scratching) were excluded from analysis. These artifacts can be easily identified based on monitoring the animal’s behavior, voltage amplitude, and spectral frequency.

### Behavioral Testing

Thermal hyperalgesia of the hindpaw was assessed by measuring the latency of the withdrawal reflex in response to a radiant heat source (Paw withdrawal stimulator, Department of Anesthesiology, University of California, SD). Individual animals were placed in a Plexiglas box on an elevated glass plate under which a radiant heat source (4.7 A) was applied to the plantar surface of the hind paw after 15-minute acclimation. Paw withdrawal latencies (PWL) in response to 4 thermal stimulations, separated by 1 minute of rest, were averaged for each paw.

### Data Availability

The datasets generated during and/or analyzed during the current study are available from the corresponding author on reasonable request.
